# The Lack of Amyloidogenic Activity Is Persistent in Old WT and APP_swe_/PS1ΔE9 Mouse Retinae

**DOI:** 10.3390/ijms222111344

**Published:** 2021-10-20

**Authors:** Sandrine Joly, Léa Rodriguez, Vincent Pernet

**Affiliations:** 1 Centre de Recherche du CHU de Québec-Université Laval and Department of Molecular Medicine, Faculty of Medicine, Université Laval, Quebec, QC G1V 4G2, Canada; sandrine.joly@crchudequebec.ulaval.ca (S.J.); lea.rodriguez.1@ulaval.ca (L.R.); 2 Department of Ophthalmology, Inselspital, Bern University Hospital, University of Bern, 3010 Bern, Switzerland; 3 Department of Neurology, Inselspital, Bern University Hospital, University of Bern, 3010 Bern, Switzerland

**Keywords:** Alzheimer’s disease, retina, aging, electroretinogram, photoreceptors

## Abstract

We have previously reported that vision decline was not associated with amyloidogenesis processing in aging C57BL/6J wild-type (WT) mice and in a mouse model of Alzheimer’s disease, the APP_swe_/PS1ΔE9 transgenic mouse model (APP/PS1). This conclusion was drawn using middle-aged (10–13 months old) mice. Here, we hypothesized that compared with hippocampal and cortical neurons, the weak amyloidogenic activity of retinal neurons may result in a detectable release of amyloid β (Aβ) only in aged mice, i.e., between 14 and 24 months of age. The aim of the present study was thus to follow potential activity changes in the amyloidogenic and nonamyloidogenic pathways of young (4 months) and old (20–24 months) WT and APP/PS1 mice. Our results showed that in spite of retinal activity loss reported by electroretinogram (ERG) recordings, the level of amyloid beta precursor protein (APP) and its derivatives did not significantly vary in the eyes of old vs. young mice. Strikingly, the ectopic expression of human APP_swe_ in APP/PS1 mice did not allow us to detect Aβ monomers at 23 months. In contrast, Aβ was observed in hippocampal and cortical tissues at this age but not at 4 months of life. In contrast, optic nerve transection-induced retinal ganglion cell injury significantly affected the level of retinal APP and the secretion of soluble APP alpha in the vitreous. Collectively, these results suggest that the amyloidogenic and nonamyloidogenic pathways are not involved in visual function decline in aging mice. In WT and APP/PS1 mice, it is proposed that retinal neurons do not have the capacity to secrete Aβ in contrast with other cortical and hippocampal neurons.

## 1. Introduction

The non-pathological decline in vision during normal aging [1] is associated with decreased retinal cell function. Reduced retinal cell responsiveness to light stimulation can be followed non-invasively by electroretinogram (ERG) recordings in humans [2,3,4] and rodents [5,6,7]. In rodents, the loss of retinal activity may be due to moderate neuronal cell death [8,9,10] and aberrant synaptic remodeling [11,12,13]. However, the molecular mechanisms underlying retinal activity loss in normal aging are incompletely understood. In this context, we proposed to study the role of amyloid β (Aβ), which has been extensively studied in Alzheimer’s disease. 

As in other CNS areas, retinal neurons express amyloid beta precursor protein (APP), whose expression in Alzheimer’s disease patients has been linked to retinal ganglion cell (RGC) death [14]. However, using the APP_swe_/PS1ΔE9 transgenic mouse model (APP/PS1), a classical model of Alzheimer’s disease expressing human APP_swe_ that can readily be cleaved into Aβ in the hippocampus and cortex of mice [15], we were not able to detect Aβ monomers in retinal lysates at 5.5, 6 and 13 months of life [8]. Based on the analysis of APP-derived proteins, we suggested that nonamyloidogenic processing of APP may prevent Aβ release in the mouse retina. The predominant secretion of non-pathological APP-derived proteins is also suggested with the observation of soluble APP alpha (sAPPα) in the vitreous of bovine and mouse eyes [16]. Soluble APPα is thought to stimulate synaptic plasticity [17,18], neuroprotection and neurite outgrowth [19]. Its function may therefore exert beneficial effects on retinal cells during aging, as suggested in our previous study [8]. Importantly, although much less active in retinal than in brain neurons, one cannot rule out the possibility that the amyloidogenic pathway is induced in old animals, i.e., at ages ranging from 14 to 24 months of life in mice [20]. Age-dependent secretion of Aβ may vary between CNS structures. Compared with brain structures, such as the hippocampus and the cortex [21,22,23], the time course of Aβ synthesis may be shifted to older ages in the mouse retina. We therefore proposed to determine in the current study whether the amyloidogenic pathway is activated in old mice, i.e., at 20–24 months of age [20]. In addition, mouse APP and mutant human APP_swe_ may be differently processed in the retina [24]. We thus observed the APP products resulting from amyloidogenic and nonamyloidogenic processing in WT and APP/PS1 mice by Western blotting.

## 2. Results

### 2.1. Retinal Cell Activity Decrease in Old Mice

**Figure 1:**
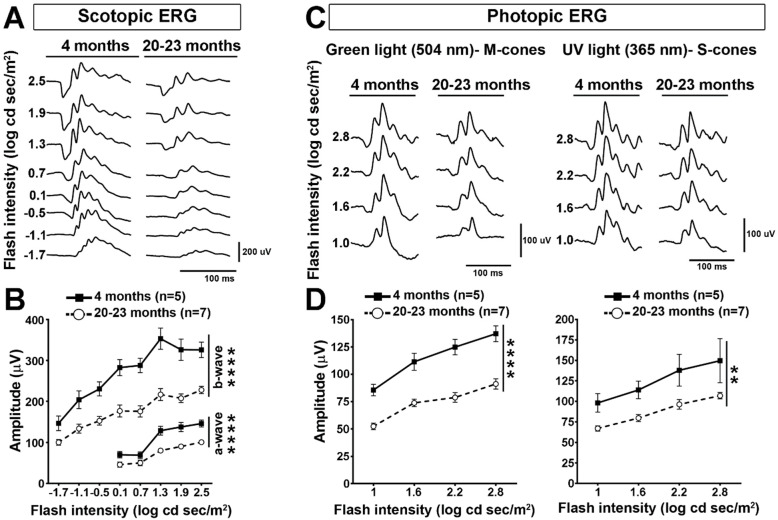
The retinal function decline in aging mice. Scotopic and photopic electroretinograms (ERG) were recorded at 4 and 23 months of age in C57BL/6J WT mice. (**A**,**B**) Scotopic ERG responses were weaker in old than in young mice for different intensities of light stimulation. Representative recordings are shown. Luminance–response curves were plotted for 5 young and 7 old mice. The amplitude of ERG a- and b-waves was statistically lower in old than in young mice (two-way ANOVA, Tukey post hoc test, ****: *p* < 0.0001). (**C**,**D**) In photopic conditions, M-cone- and S-cone-mediated ERGs were recorded with green and UV light flashes at 504 nm and 365 nm, respectively. Changes in b-wave amplitudes were measured and presented for different light intensities in luminance–response curves. The decreased b-wave amplitude revealed a significant loss of retinal activity after the stimulation of M-cones and S-cones with various intensities of light in old mice. The M-cone pathway turned out to be more affected by aging than that depending on S-cones (two-way ANOVA, Tukey post hoc test; **: *p* < 0.01, ****: *p* < 0.0001).

In this study, we wondered if the endogenous expression of APP and its transformation into Aβ may be responsible for age-associated retinal function decline. In order to determine the effect of aging on retinal function, we first recorded ERGs in mice at 4 months and 20–23 months of age (Figure 1). In scotopic conditions (dark background), the ERG response of 23-month-old mice was weaker for a large range of light stimulation intensities than that of younger mice (4 months) (Figure 1A). Quantitatively, the amplitudes of the a-wave, resulting from the mixed response of rod and cone photoreceptors, and of the b-wave, generated by postreceptoral cells (bipolar cells and Müller glia) were significantly decreased (Figure 1B). In photopic conditions as well, the ERG amplitude was significantly lower in old than in young mice (Figure 1C). Interestingly, the ERG response was more decreased with light stimulation at 504 nm than at 365 nm, suggesting that the M-cone pathway is more strongly affected by aging than that mediated by S-cones (Figure 1D). In general, these data show a strong loss of ERG response at 23 months of age compared with our previous observations led in 13-month-old animals [8]. 

### 2.2. Endogenous Processing of Mouse Amyloid β Precursor Protein in Aged Animals

**Figure 2:**
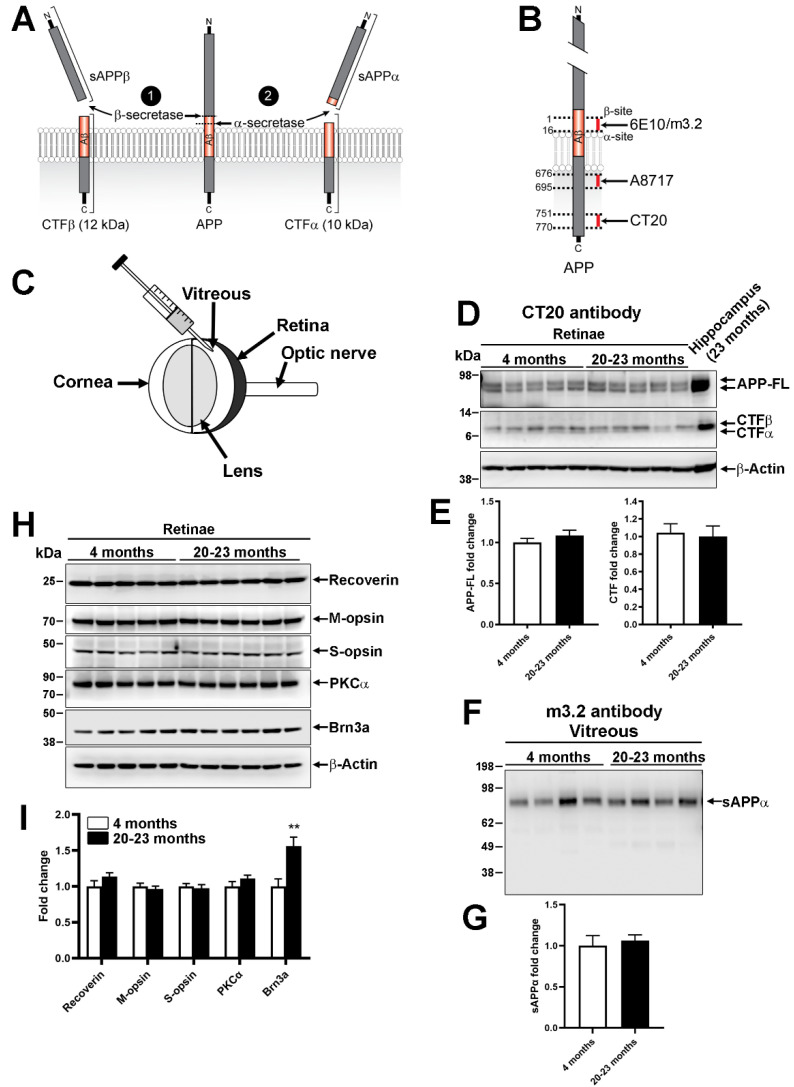
Expression of constitutive retinal proteins and amyloid precursor protein products in old vs. young WT mice. (**A**) Scheme showing possible amyloid precursor protein (APP) processing in neurons. (**B**) APP epitopes recognized by antibodies in Western blot analyses. (**C**) Mouse eye anatomy. The retina and vitreous were separately processed for APP fragment detection by Western blotting. (**D**,**E**) Detection of full-length APP (APP-FL) and C-term fragment alpha (CTFα) and beta (CTFβ) in retinal lysates. Five animals per age were examined. Densitometric quantifications of bands in ImageJ software (NIH) did not reveal protein expression change between the two age groups. (**F**,**G**) sAPPα release in 4 μL of mouse vitreous was analyzed in the same mice. Quantitative analysis did not show significant difference. (**H**,**I**) The level of proteins expressed in the different retinal cell layers did not significantly vary along with age except that of Brn3a, which was statistically higher in old retinae than in young ones (unpaired *t*-test, **: *p* < 0.01). Cartoons presented in (**A**,**B**) are from [8].

In the so-called amyloidogenic pathway, the activation of β-secretase can induce the cleavage of APP into toxic Aβ and C-terminal fragment β (CTFβ), whereas the nonamyloidogenic transformation mediated by α-secretases results in the shedding of non-toxic protein fragments, such as sAPPα and transmembrane CTFα (Figure 2A). We sought to determine if the potential release of Aβ may coincide with the reduction in ERG activity observed in old mice (Figure 1). Using CT20 antibody (Figure 2B), the expression level of APP and its CTF products were followed by Western blotting in retinal lysates from young (4 months) and old mice (20–23 months) (Figure 2D). We observed that the retinal level of full-length APP (APP-FL) did not vary much between young and old mice (Figure 2D,E). Interestingly, CTFβ and CTFα were present at similar levels in young and old retinal samples. Proteins from the vitreous body were separately analyzed (Figure 2F,G). Indeed, the vitreous has been shown to contain APP-derived proteins, such as sAPPα or Aβ, shed from the surface of retinal neurons [16]. In addition, sAPPα detection with m3.2 antibody showed no difference in aged vs. young retinae (Figure 2F,G). The lack of change for intravitreal sAPPα and retinal CTFβ suggests that the amyloidogenic and nonamyloidogenic pathways are not influenced by aging. Moreover, the level of proteins specifically expressed in different retinal cell subpopulations did not vary between old and young mice, with the exception of Brn3a, a marker of RGCs, whose level was statistically higher in old retinae (Figure 2H,I). 

### 2.3. Human Amyloid β Precursor Protein Transformation in Transgenic APP/PS1

**Figure 3:**
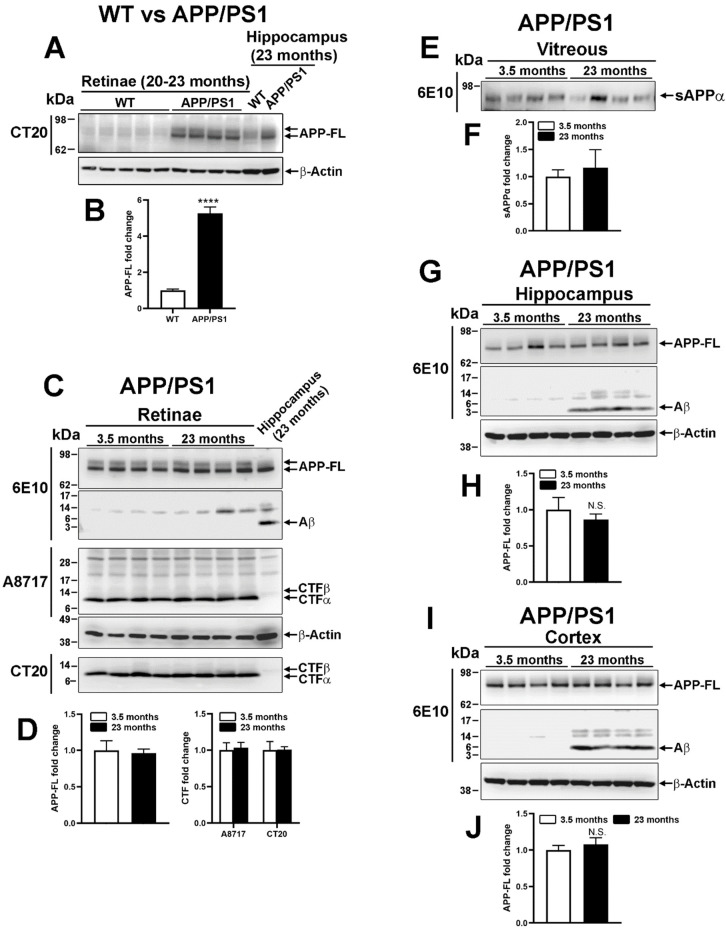
Analysis of amyloidogenic processing in very old APP/PS1 mouse retinae. (**A**,**B**) Western blotting analysis showed stronger expression of full-length APP (APP-FL) in the retinae of APP/PS1 transgenic mice expressing human APP than in WT samples (unpaired *t*-test, ****: *p* < 0.0001). The level of APP-FL was similar to that observed in the hippocampus. (**C**,**D**) The level of human APP was similar in young and old APP/PS1 retinae. However, Aβ monomer was only detectable in the hippocampus. CTFα was predominantly expressed in the retina, with no change associated with age, although CTβ was observed in hippocampal homogenate, suggesting predominant nonamyloidogenic processing of human APP in the mouse retina. (**E**,**F**) In very old APP/PS1 mice, the intravitreal level of sAPPα did not differ from that of young animals. (**G**–**J**) Age-dependent increased synthesis of Aβ monomer was observed in the hippocampus and cortex of APP/PS1 mice. However, the level of APP-FL did not change in an age-dependent manner.

The ectopic expression of human APP_swe_ in APP/PS1 mice allows us to observe Aβ deposits in the hippocampus and cortex of mice from 9 months of age [21]. APP_swe_ is a mutant protein readily converted by β-secretase into Aβ and CTFβ. APP-derived proteins were thus analyzed by Western blotting in WT and APP/PS1 retinae at 20–23 months of age (Figure 3A,B). Using CT20 antibody (Figure 2B), APP-FL appeared strongly upregulated in the retina of APP/PS1 mice compared with WT mice (Figure 3A,B). Contrary to mouse APP, whose level is endogenously higher in the hippocampus than in the retina of WT animals (Figure 2D), human APP was expressed at similar levels in the hippocampus and in the retina of APP/PS1 mice (Figure 3A). However, soluble Aβ monomers could not be observed at 3.5 and 23 months of age in APP/PS1 retinae in contrast to the hippocampus (Figure 3C,D). Moreover, CTFα was predominantly expressed over CTFβ in APP/PS1 retinae (Figure 3C,D), independently of age. The intravitreal content of sAPPα did not significantly change in old eyes when compared with young ones (Figure 3E,F). In comparison to the retina, the hippocampus (Figure 3G,H) and the cortex (Figure 3I,J) from the same APP/PS1 mice contained Aβ at 23 months but not at 3.5 months of age, thus demonstrating the influence of aging on the production of Aβ in brain tissues. 

### 2.4. The Expression and the Nonamyloidogenic Processing of APP Are Influenced by Optic Nerve Injury

**Figure 4:**
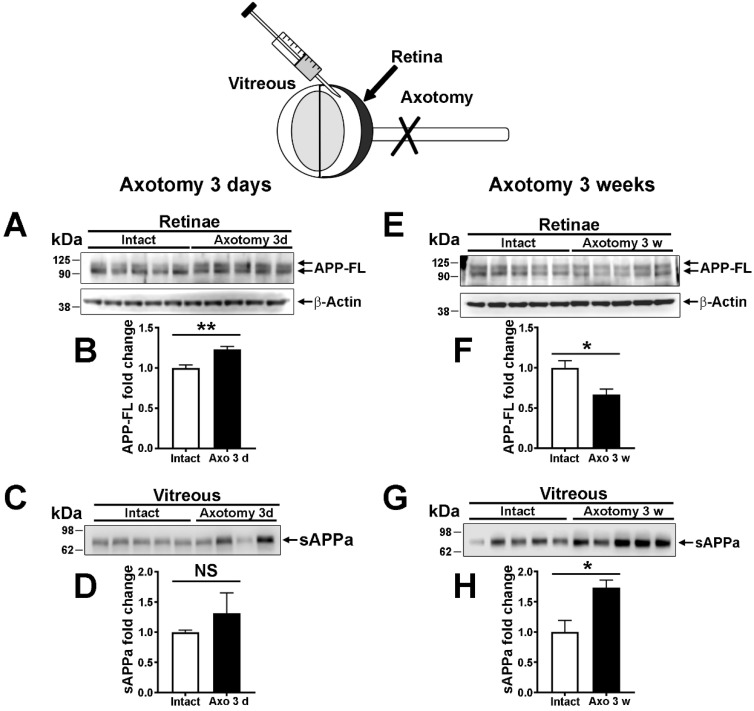
Nonamyloidogenic transformation of APP in the retina does not depend on retinal ganglion cells. The optic nerve injury paradigm was used to selectively eliminate retinal ganglion cells (RGCs) in WT retinae. Vitreous samples were withdrawn from the eyeball under anesthesia using a 10-μL Hamilton syringe. (**A**,**B**) Three days after optic nerve injury, a time when injured RGCs are not yet lost, APP-FL was significantly upregulated in retina lysates compared with intact conditions (unpaired *t*-test, **: *p* < 0.01). (**C**,**D**) In the vitreous, sAPPα was not significantly increased (NS). (**E**,**F**) Three weeks post-injury, the level of APP-FL was significantly decreased in lesioned retinae compared with intact ones (unpaired *t*-test, *: *p* < 0.05). (**G**,**H**) At the same age, the level of sAPPα was enhanced in the vitreous of injured mice (unpaired *t*-test, *: *p* < 0.05), suggesting that nonamyloidogenic products were secreted by α-secretase independently of RGCs.

In the mouse retina, RGCs are thought to represent an important source of APP. To determine their contribution to retinal APP expression, RGCs were selectively eliminated using complete optic nerve injury (Figure 4). Optic nerve axon transection induces retrograde degeneration and apoptotic cell death of RGCs [25]. In this lesion model, the loss of RGCs only starts 5 days post-injury [26]. Three weeks after optic nerve injury, less than 10% of RGCs remain alive [27]. We thus examined the level of APP and its derivatives 3 days and 3 weeks post-injury. Retinal APP-FL was upregulated 3 days after optic nerve lesion, a time associated with the injury response of RGCs (Figure 4A,B). At the same time, although abundant, sAPPα did not significantly vary in the vitreous in response to optic nerve injury (Figure 4C,D). In contrast, retinal APP-FL was significantly decreased at 3 weeks, when the majority of RGCs are dead (Figure 4E,F). In the vitreous body, the level of sAPPα increased in injured eyes relative to intact mice (Figure 4G,H). Together, these results indicate that APP-FL expression increases early in the injury response of RGCs, whereas a rise in intravitreal sAPPα seems to be sustained when the majority of RGCs are lost.

## 3. Discussion

Our previous study revealed the lack of amyloidogenic products in the retina of middle-aged WT and APP/PS1 mice at 10–13 months of age. In the present study, we wondered if the amyloidogenic release of proteins such as Aβ may arise in older mice. In brain structures susceptible to the formation of senile plaques, Aβ aggregates accumulate between 10 and 24 months, an age that is considered as old [28]. By using old mice, we report here that the expression of mouse APP remains stable in the retina compared with young adults. In addition, the level of APP fragments released by the amyloidogenic and nonamyloidogenic pathways in WT and APP/PS1 mice was not influenced by the age of mice. Our data demonstrate that contrary to the hippocampus and the cerebral cortex, the retina does not possess the ability to produce Aβ in old mice that exhibit marked visual function decline due to aging. In addition, optic nerve injury-induced RGC depletion showed that APP and sAPPα levels can be modulated by neuronal injury and are produced in great part by other retinal cells. 

### 3.1. Visual Function Decrease in Old Mice

In the present study, we observed that the scotopic ERG response generated by photoreceptors and postreceptoral cells was deeply affected by the age of mice. The decrease in ERG activity recorded in 20–24-month-old mice was stronger than that previously reported in those 10–13 month of age [8]. Strikingly, however, the level of cell-specific retinal cell markers, namely, recoverin and opsins for photoreceptors, PKCα for bipolar cells and Brn3a for RGCs, did not decrease in old vs. young retinae. This suggests that cell death is not the principal factor accounting for the loss of retinal activity in the outer retina. Consistently, histological examinations in other studies showed that retinal cell survival is weakly affected in old mice [8,29]. However, retinal cell subpopulations may undergo cell death at a moderate rate in the inner retina, as previously shown by Samuel et al. [9]. In fact, cell death in the inner retina is not expected to affect the function of photoreceptors. Aberrant neurite outgrowth and synaptic remodeling in the aging retina can disturb neurotransmission between photoreceptors and postsynaptic neurons in the inner nuclear cell layer and, hence, reduce ERG function [11,12,13,30]. Interestingly, photopic ERG recordings revealed that the M-cone pathway was significantly more affected than that of S-cones in old mice. This difference has not been observed in middle-aged mice before [8]. The fact that M-opsin and S-opsin protein expressions did not change in old mice (present study) indicate that the decreased response obtained in photopic conditions is unlikely due to the regulatory mechanisms of phototransduction, such as those involving cone photopigment expression changes [31]. In old mice, a fraction of M-cones that are localized in the peripheral retina may be more susceptible to death than S-cones, according to Cunea and colleagues [10]. The identification of the molecular mechanisms contributing to retinal function loss may allow the improvement of sight in normally aging individuals.

### 3.2. Amyloidogenic vs. Nonamyloidogenic Processing in the Mouse Retina

The high level of sAPPα in the vitreous and CTFα in the retina suggests that the nonamyloidogenic transformation of APP in APP/PS1 mice may limit the release of toxic Aβ, similarly to what we have observed in middle-aged animals [8]. Indeed, the cleavage of the APP substrate into α fragments prevents its enzymatic cut by β-secretases. In addition, our new results obtained in old mice allow us to rule out the potential late secretion of Aβ. For neurons that are prone to secrete Aβ in hippocampal and cortical tissues, the amount of Aβ was significantly increased at 24 months but not at 4 months of life. However, we cannot exclude the possibility that Aβ may be synthesized at low, sublethal concentrations in the mouse retina. Indeed, the detection of CTFβ suggests that β-secretases are active in young and old WT retinae. At a low level, Aβ has been found to be involved in memory formation and in synapse plasticity [32,33]. It may also fulfill an important function in retinal neurotransmission in physiological conditions. Moreover, a potential toxic effect of Aβ may be avoided in the retina by its rapid efflux through the glymphatic drainage system of the eye [34]. Overall, the fact that Aβ is detected in brain structures but not in the retina of APP/PS1 mice strongly supports the notion that retinal neurons possess an intrinsic weak ability to produce Aβ, independently of aging. This phenomenon may constitute a natural mechanism of neuroprotection that may be used in new therapies to counteract Aβ neurotoxicity in vulnerable brain regions. 

### 3.3. Modulation of Retinal APP Expression and Secretion of sAPPα in the Vitreous after Lesion

The decrease in retinal APP by ~40%, 3 weeks post-injury, suggests that RGCs are a relatively important source of APP in the retina. Indeed, this percentage is proportionally high for RGCs that only represent ~0.5–1% of total retinal cells. We have previously observed APP expression in other retinal layers [8]. Following axotomy-induced RGC depletion, retinal cells, such as amacrine cells and bipolar cells, may continue to express APP. Surprisingly, the intravitreal level of sAPPα significantly increased at 3 weeks but not at 3 days post-lesion. This time-dependent change suggests that the activation of α-secretase occurs when the bulk of RGCs is lost. The role of sAPPα in the injured retina is not known. We speculate that sAPPα upregulation may be part of endogenous mechanisms aiming at promoting retinal cell rewiring after RGC depletion. Indeed, adenovirus-mediated sAPPα expression has previously been shown to enhance synaptic function in the hippocampus of APP/PS1 mice [17,35,36]. In addition, sAPPα can act as a potent growth-promoting agent in neuronal cell cultures [37,38]. In the injured retina, deprived of RGCs, sAPPα might thus increase to stimulate neuronal rewiring, although its late rise cannot induce plastic mechanisms compensating for the massive elimination of RGCs. To clarify the function of sAPPα, it would be interesting to follow retinal function changes and new synapse formation after intravitreal injection of recombinant sAPPα early after optic nerve injury. In contrast, the upregulation of APP that was observed 3 days after optic nerve injury may be involved in optic nerve injury-induced cell death. Indeed, Liu and colleagues found a quick and transient increase in APP expression in axotomized RGCs [39]. The deletion of APP in knockout mice allowed an increase in the survival of injured RGCs by downregulating Jun kinase 3 (JNK3) activation [39]. Based on these studies, the antagonistic effects of APP-FL and sAPPα on neuronal survival and neuroplasticity may be modulated to improve vision in diseases associated with optic nerve injury, such as glaucoma [40]. 

In conclusion, our data confirm that the amyloidogenic pathway, controlling the secretion of neurotoxic Aβ in Alzheimer’s disease, is not activated in old retinal neurons. This conclusion has two possible implications: (1) the molecular mechanisms responsible for age-associated visual decline do not depend on Aβ and APP in the mouse eye, and (2) the elucidation of the mechanisms preventing retinal amyloidogenesis might be used to protect brain structures readily producing Aβ in Alzheimer’s disease. 

## 4. Materials and Methods

### 4.1. Animals

The experiments were performed in young (3.5–4 months of age) and old (23 months of age) male wild-type (WT) and APP_swe_/PS1ΔE9 mice maintained in a C57BL/6J background. Transgenic APP_swe_/PS1ΔE9 mice were originally purchased from the Jackson Laboratory (Bar Harbor, ME, USA). Animals were genotyped as previously described [41]. 

### 4.2. Electroretinography

Electroretinograms (ERGs) were recorded in young (n = 5) and old (n = 7) C57BL/6J WT mice with a Ganzfeld ERG system (Phoenix Research Labs, Pleasanton, CA, USA), as previously reported [8,29]. Briefly, mice were adapted to complete darkness for 12 h beforehand for scotopic recordings. Under dim red light, mice were anesthetized with an intraperitoneal injection of a ketamine–xylazine (100–10 mg/kg) mixture. After pupil dilation (1% Mydriacyl Tropicamide, Alcon, Geneva, Switzerland) and corneal hydration (Tear-Gel, Baush & Lomb, New York, NY, USA), mice were placed on a platform, and the reference and ground electrodes (platinum needles) were subcutaneously inserted on top of the head and into the tail, respectively. Scotopic full-field ERGs (bandwidth: 2–1000 Hz) were obtained in response to increasing flash intensities, ranging from −1.7 log cd.s.m^−2^ to 2.5 log cd.s.m^−2^ (interstimulus interval, 20 msec; flash duration, 1 msec). The mouse eye was then exposed to constant green light (504 nm; 1.6 log cd/m^2^) for 10 min in order to saturate rod photoreceptors to record photopic ERGs. Green light flashes (504 nm) and UV light flashes (365 nm) were sequentially sent (interstimulus interval, 20 msec; flash duration, 1 msec; average of 20 flashes, from 0.1 to 2.8 log cd.s.m^−2^; 0.6 log-unit increment) to selectively excite the M-cone and the S-cone pathways. 

For scotopic ERG analysis, the amplitude of the a-wave was measured from baseline to the most negative trough, while that of the b-wave was measured from the trough of the a-wave to the most positive peak of the retinal response [42]. Scotopic luminance–response function curves were obtained by plotting the amplitude of the b-wave as a function of the flash intensity used to evoke the response. Statistical analysis was performed by a one-way ANOVA with Tukey post hoc test (GraphPad Prism, GraphPad Software, La Jolla, CA, USA).

### 4.3. Western Blot Analysis

Under deep anesthesia, mouse vitreous was carefully aspirated from both eyes using a 10-μL Hamilton syringe mounted with a glass-tipped needle. Vitreous samples were immediately flash frozen in liquid nitrogen and stored at −80 °C. Mice were then intracardially perfused with PBS to eliminate blood cells from tissue samples. The retinae and hippocampi of each mouse were quickly dissected, placed in an Eppendorf tube, snap frozen in liquid nitrogen and stored at −80 °C until protein lysate preparation. For cortical homogenates, a piece of ~1 mm^2^ of cortex was cut with a scalpel blade after skull opening. Samples were homogenized for 60 min on ice in Eppendorf tubes containing lysis buffer (20 mM Tris-HCl, 0.5% CHAPS, pH 8.0) and protease/phosphatase inhibitor (Roche Diagnostics, Laval, QC, Canada) and centrifuged for 15 min at 15,000× *g* at 4 °C. Protein samples (20 μg/lane) were resolved by electrophoresis on a 4–12% gradient polyacrylamide gel and transferred to nitrocellulose membranes. After pre-incubation in a blocking solution of 5% BSA dissolved in TBST (Tris-base 0.1 M, 0.2% Tween 20, pH 7.4) for 1 h at room temperature, membranes were incubated with primary antibodies (see Table 1) overnight at 4 °C. After washing, the membranes were incubated with a horseradish peroxidase-conjugated anti-mouse, anti-rabbit (1:10,000–1:15,000; Pierce Biotechnology, Burlington, ON, Canada) or anti-goat antibody (1:5000; Santa Cruz, Dallas, TX, USA). Chemiluminescent bands were detected with LiCor Western Sure Premium Chemiluminescent Substrate (Mandel, Guelph, ON, Canada) in a LiCor C-Digit blot scanner (Mandel). Band signals were quantified with the ImageJ software (Rasband, W.S., ImageJ, U.S. National Institutes of Health, Bethesda, Maryland, USA, https://imagej.nih.gov/ij/, 1997–2018) and analyzed with the GraphPad Prism software.

### 4.4. Optic Nerve Injury

Intraorbital optic nerve transection (axotomy) was carried out at ~0.5 mm from the back of the left eyeball under general anesthesia using 2% isoflurane mixed in a gas flow of 2% oxygen. To relieve pain, mice received a subcutaneous injection of buprenorphine (0.2 mg/kg) 30 min before starting the surgery. In order to obtain access to the optic nerve, a suborbital incision was made with a scalpel blade. The optic nerve sheath was longitudinally cut with micro-scissors by taking care not to damage the ophthalmic artery. After transection, optic nerve stumps were placed back in the optic nerve sheath, and the skin opening was closed with 6.0 silk suture. The integrity of blood circulation was controlled in the retinae by fundoscopy right after the injury. Single animals were then put in a clean cage until complete recovery. 

## Figures and Tables

**Table 1:** Antibodies used for Western blot analysis.

Name	Species	Dilution	Source	Catalog Number
β-Actin	mouse	1:5000	Sigma, Oakville, ON, Canada	A5441
β-Amyloid (m3.2)	mouse	1:1000	BioLegend, San Diego, CA, USA	805701
β-Amyloid (6E10)	mouse	1:2000	BioLegend	803001
APP C-Term (CT20)	rabbit	1:10,000	Millipore, Etobicoke, ON, Canada	171610
Amyloid Precursor Protein, C-Term	rabbit	1:5000	Sigma	A8717
Brn3a	mouse	1:200	Santa Cruz	sc-8429
M-opsin	rabbit	1:5000	Millipore	AB5405
PKCα	mouse	1:200	Santa Cruz	sc-17769
Recoverin	rabbit	1:2000	Millipore	AB5585
S-opsin	goat	1:2000	Santa Cruz	sc-14363
